# Supplementary material for: Dynamical network analysis reveals key microRNAs in progressive stages of lung cancer
Source: PLoS Comput Biol. 2020 May 19;16(5):e1007793. doi: 10.1371/journal.pcbi.1007793 (PMC7295246; doi:10.1371/journal.pcbi.1007793)
Supplement: S1 Appendix — (PDF) [file pcbi.1007793.s001.pdf]

S1 Appendix

TNM stages

Lung Adenocarcinoma (LUAD) is a kind of lung cancer that is a malignant tumor. The International Association for the Study of Lung Cancer established the Lung Cancer Staging Project in 1998 and updated the Tumor-Node-Metastasis (TNM) classification system of malignant tumors, which forms the basis for classifying lung cancer into distinct stages. The current (seventh) edition classifies all histotypes of lung cancer after evaluation of the outcomes from an extensive worldwide database of patients.

TNM characterizes the size and location of the tumor, the location of cancer in the lymph nodes and to where the cancer has spread (Metastases). In particular, the letter "T" stands for primary tumor:

"T1a" – tumor size $\leq$ 2cm;

"T1b" – tumor size $> 2 \sim 3$cm;

"T2a" – tumor size $> 3 \sim 5$cm;

"T2b" – tumor size $> 5 \sim 7$cm;

"T3" – tumor size $>$ 7cm and/or multiple tumor nodules in the same lobe;

"T4" – multiple tumor nodules (of any size) in the same lung but in a different lobe.

The letter "N" denotes the spread of the cancer to nearby lymph nodes:

"N0" – no regional lymph node metastasis;

"N1" – metastasis in ipsilateral peribronchial and/or ipsilateral hilar lymph nodes as well as intrapulmonary nodes including involvement by direct extension;

"N2" – metastasis in ipsilateral mediastinal and/or subcarinal lymph node(s);

"N3" – metastasis in contralateral mediastinal, contralateral hilar, ipsilateral or contralateral scalene, or supraclavicular lymph node(s).

The letter "M" describes distant metastasis:

"M1a" – malignant pleural or pericardial effusions and/or separate tumor nodules in the contralateral lung;

"M2b" – distant metastasis in extrathoracic organs.

Based on the seventh edition of the TNM classification system described above, we list the stage groupings for non-small-cell lung cancer in the table below. In order to have sufficient data amount for statistical analysis of each LUAD stage, in our work we combine stage Ia and Ib and label them as stage I, and do the same for Stages II, III, and IV.

Table. Stage groupings for non-small-cell lung cancer.

	Tumour (T)	Node (N)	Metastasis (M)
Ia	T1a or T1b	N0	M0
Ib	T2a	N0	M0
IIa	T1a or T1b	N1	M0
	T2a	N1	M0
	T2b	N0	M0
IIb	T2b	N1	M0
	T3	N0	M0
IIIa	T1 or T2	N2	M0
	T3	N1 or N2	M0
	T4	N0 or N1	M0
IIIb	T4	N2	M0
	any T	N3	M0
IV	any T	any N	M1a
	any T	any N	M1b
