# Supplementary material for: Dynamical network analysis reveals key microRNAs in progressive stages of lung cancer
Source: PLoS Comput Biol. 2020 May 19;16(5):e1007793. doi: 10.1371/journal.pcbi.1007793 (PMC7295246; doi:10.1371/journal.pcbi.1007793)
Supplement: S2 Appendix — We have used four methods of statistical analysis involving hypothesis testing to calculate the P-values. (PDF) [file pcbi.1007793.s002.pdf]

S2 Appendix

Statistical analysis and P-value

We have used four methods of statistical analysis involving hypothesis testing to calculate the P-values. The four methods are as follows.

- In our construction of the CeRNA network, RNAs are processed through a differentially expressed analysis, where the basic statistic used for significance assessment is the moderated t-statistic [1], which is computed for each contrast. This has the same interpretation as an ordinary t-statistic except that the standard errors have been moderated across genes, i.e., squeezed towards a common value, using a simple Bayesian model. This has the effect of borrowing information from the ensemble of genes to enhance inference about each individual gene. Moderated t-statistics lead to P-values in the same way that ordinary t-statistics do except that the degrees of freedom are increased, reflecting the greater reliability associated with the smoothed standard errors.

- The two-sample Wilcoxon test (also known as "Mann-Whitney" test) [2, 3] is applied to vectors of the RNA expression data, with the following notations for statistical significance: ns – p > 0.05; * – p ≤ 0.05; ** – p ≤ 0.01; *** – p ≤ 0.001; **** – p ≤ 0.0001.

- Gene enrichment analysis as described in MATERIALS AND METHODS and implemented in Figs. 3 and 5 in the main text.

- Log-rank tests as described at the end of subsection entitled "Framework of analysis" in MATERIALS AND METHODS in the main text.

References

[1] Phipson B, Lee S, Majewski IJ, Alexander WS, Smyth GK. Robust hyperparameter estimation protects against hypervariable genes and improves power to detect differential expression. Ann Appl Stat. 2016;10(2):946.

[2] Bauer DF. Constructing confidence sets using rank statistics. J Ame Stat Asso. 1972;67:687–690.

[3] Hollander M, Wolfe DA. Nonparametric Statistical Methods. New York: John Wiley & Sons; 1973.
