# Supplementary figures and images for: Dynamical network analysis reveals key microRNAs in progressive stages of lung cancer
Source: PLoS Comput Biol. 2020 May 19;16(5):e1007793. doi: 10.1371/journal.pcbi.1007793 (PMC7295246; doi:10.1371/journal.pcbi.1007793)

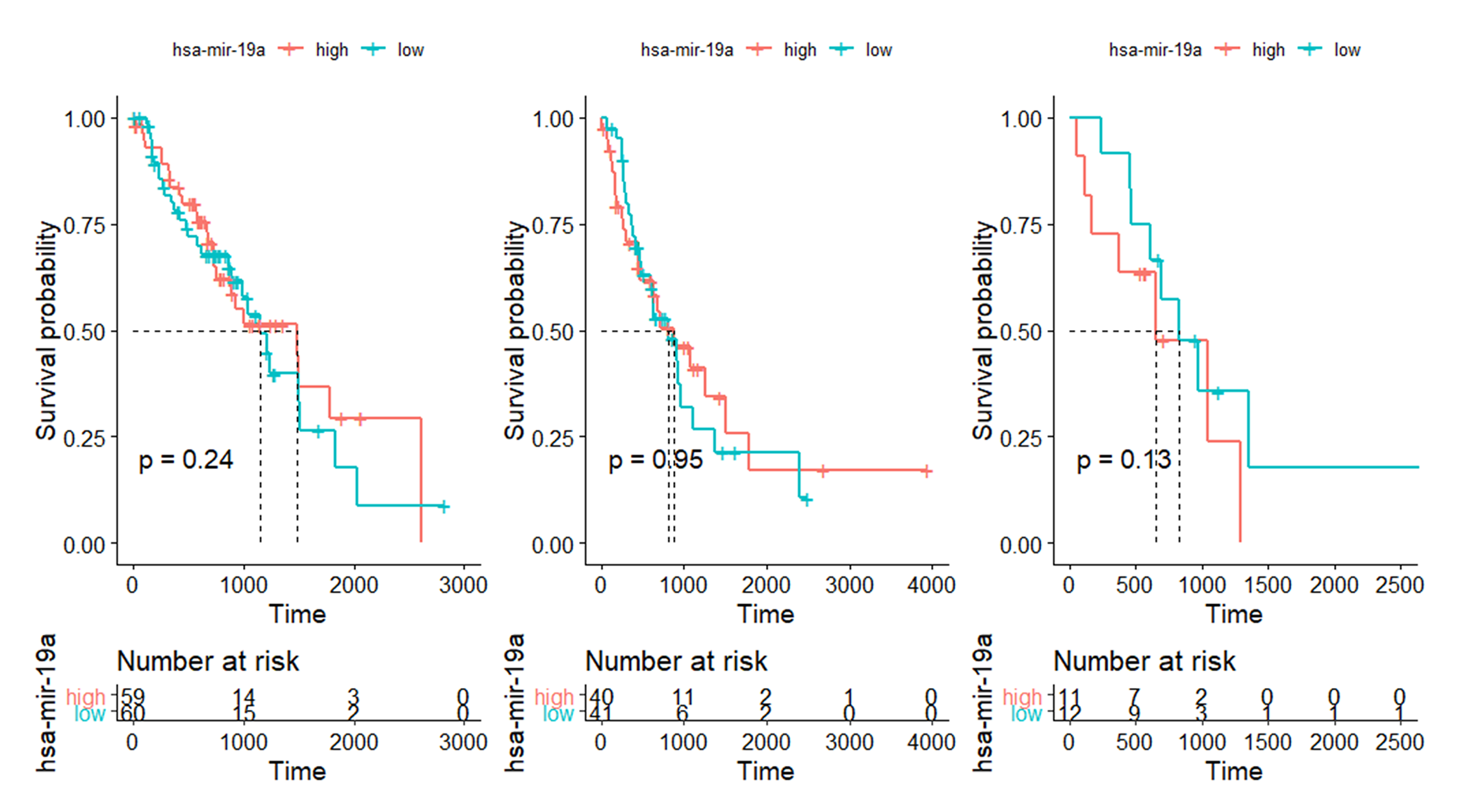

Supplement: S1 Fig — The P-values of hsa-mir-19a in the Kaplan-Meier survival curves are 0.24, 0.95, 0.13 for stages II, III and IV, respectively. Log-rank tests are used to analysis of Kaplan-Meier survival curve. (TIF) [file pcbi.1007793.s003.TIF]

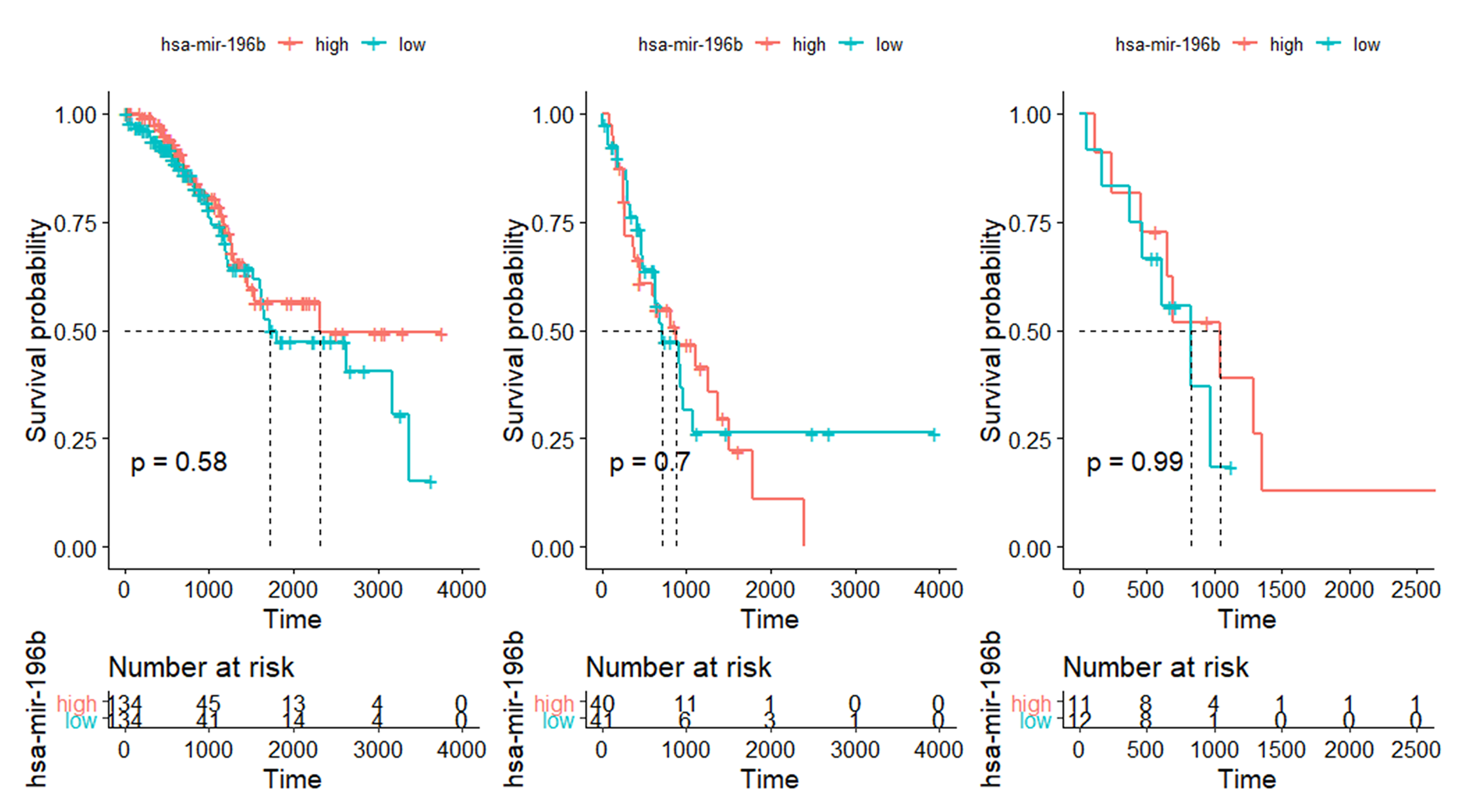

Supplement: S2 Fig — The P-values of hsa-mir-196b in the Kaplan-Meier survival curves are 0.58, 0.7, 0.99 for stages I, III and IV, respectively. (TIF) [file pcbi.1007793.s004.TIF]

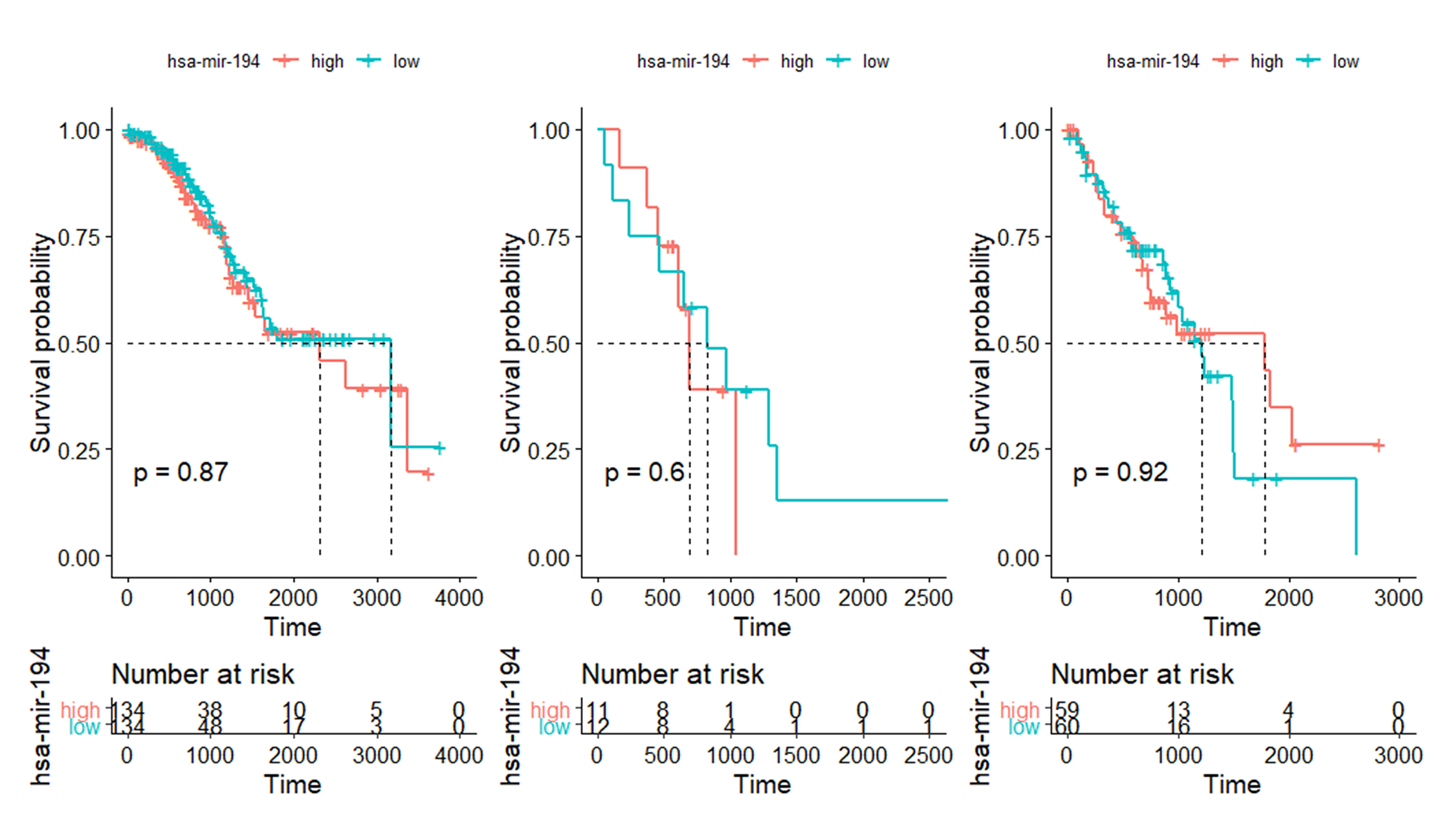

Supplement: S3 Fig — The P-values of hsa-mir-194 in the Kaplan-Meier survival curves are 0.87, 0.92, 0.6 for stages I, II and IV, respectively. (TIF) [file pcbi.1007793.s005.TIF]

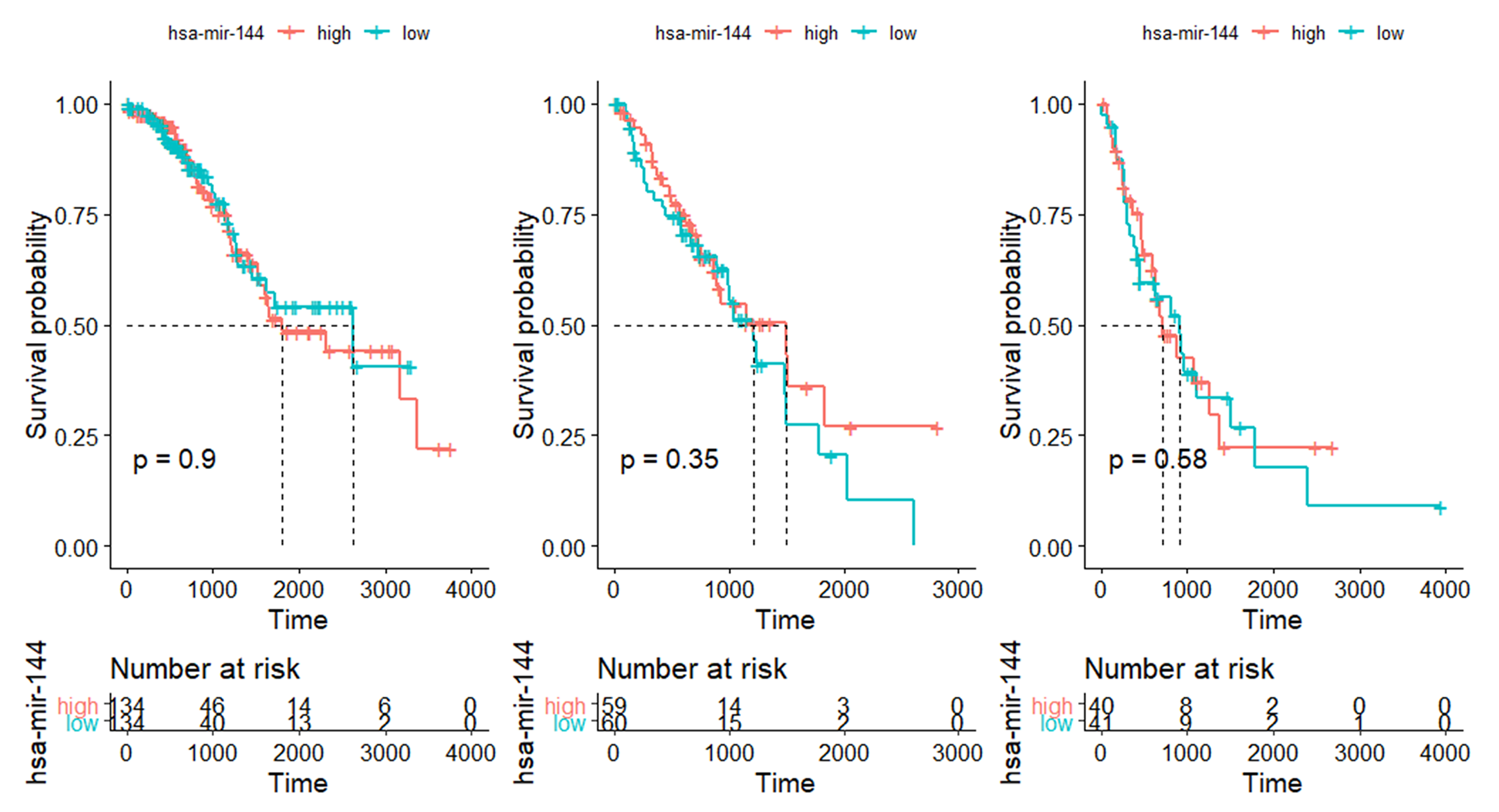

Supplement: S4 Fig — The P-values of hsa-mir-144 in the Kaplan-Meier survival curves are 0.9, 0.35, 0.58 for stages I, II and III, respectively. (TIF) [file pcbi.1007793.s006.TIF]

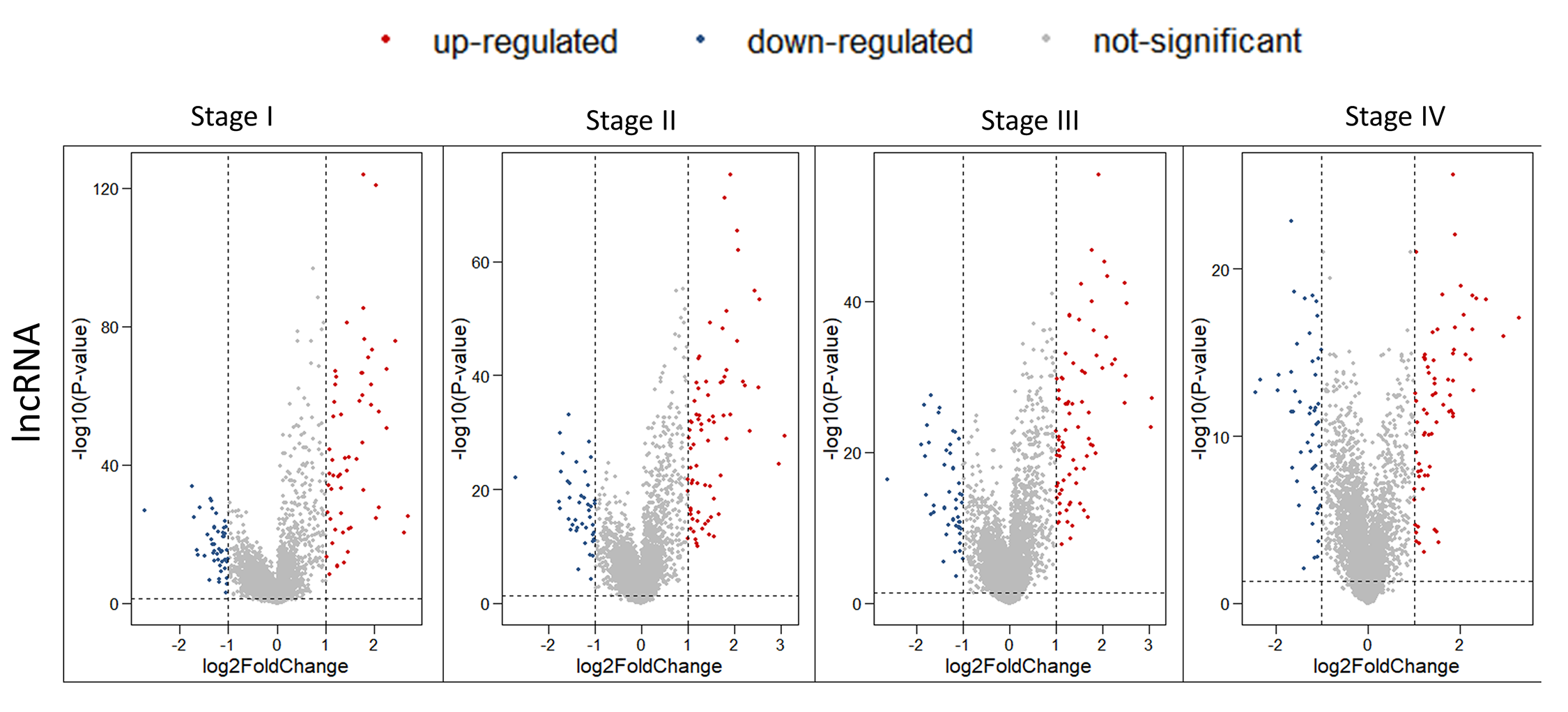

Supplement: S5 Fig — Volcano map of the lncRNA expression level of four stages of LUAD samples. The x-axis is log2 Fold−Change (FC value), and the y-axis is the P-values from the differential expression analysis. (TIF) [file pcbi.1007793.s007.TIF]

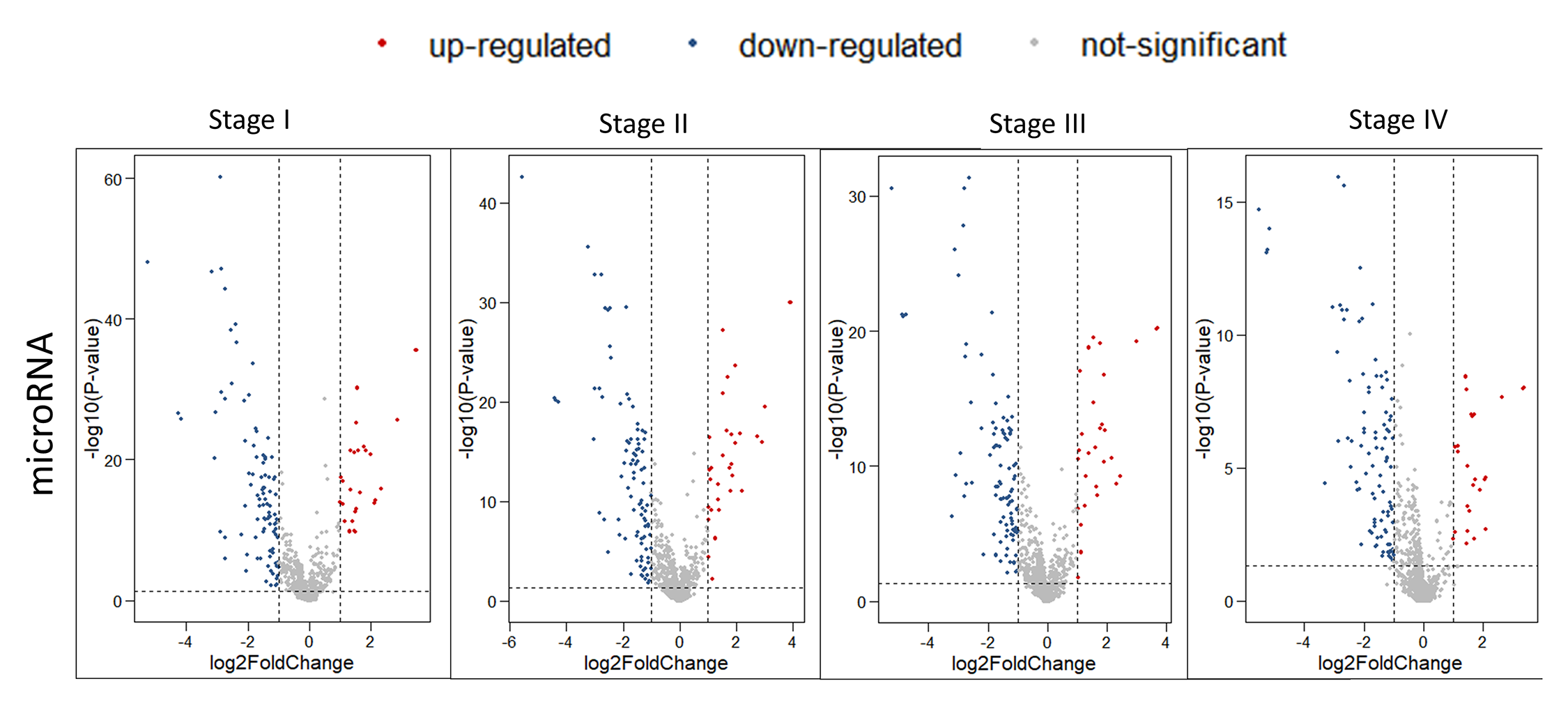

Supplement: S6 Fig — Volcano map of the microRNA expression level of four stages of LUAD samples. (TIF) [file pcbi.1007793.s008.TIF]

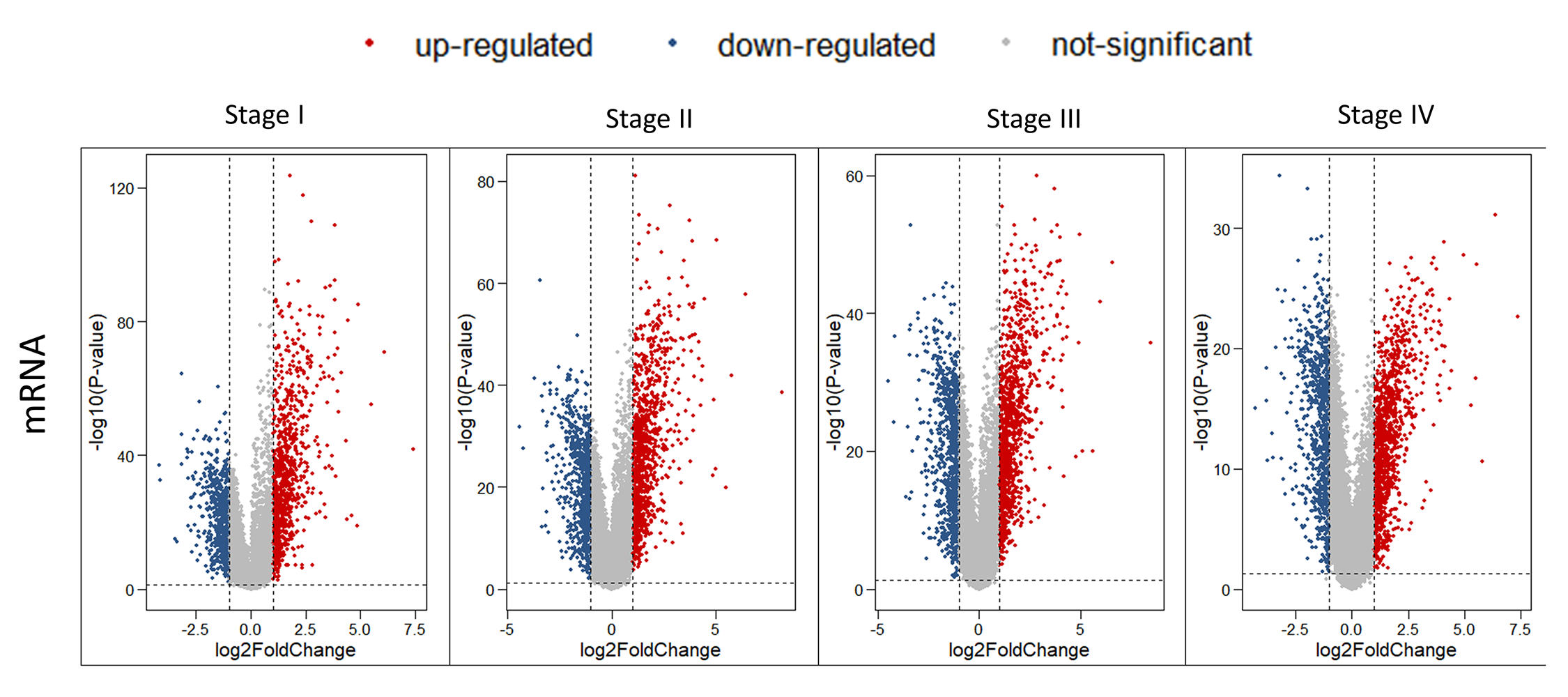

Supplement: S7 Fig — Volcano map of the mNA expression level of four stages of LUAD samples. (TIF) [file pcbi.1007793.s009.TIF]
