# Supplementary material for: Dynamical network analysis reveals key microRNAs in progressive stages of lung cancer
Source: PLoS Comput Biol. 2020 May 19;16(5):e1007793. doi: 10.1371/journal.pcbi.1007793 (PMC7295246; doi:10.1371/journal.pcbi.1007793)
Supplement: S1 Table — Detailed parameter values of the reconstructed lncRNA-microRNA-mRNA CeRNA networks. (PDF) [file pcbi.1007793.s010.pdf]

**S1 Table. Numbers of nodes and edges of CeRNA networks.**

| | lncRNA | | | microRNA | | | mRNA | | | all nodes | all edges |
|---|---|---|---|---|---|---|---|---|---|---|---|
| | total | dif | same | total | dif | same | total | dif | same | | |
| Stage I | 56 | 27 | 29 | 58 | 5 | 53 | 480 | 197 | 283 | 594 | 1633 |
| Stage II | 59 | 30 | | 55 | 2 | | 519 | 236 | | 633 | 1603 |
| Stage III | 70 | 41 | | 61 | 8 | | 595 | 312 | | 726 | 1952 |
| Stage IV | 65 | 36 | | 57 | 4 | | 618 | 335 | | 740 | 1945 |

The rows denoted as stages I, II, III, and IV correspond to the networks at the four stages of LUAD.
The columns "total," "dif," and "same" represent the total number of nodes, the number of unique nodes, and the number of common nodes, respectively, for the networks at the four stages.
