# Supplementary material for: Dynamical network analysis reveals key microRNAs in progressive stages of lung cancer
Source: PLoS Comput Biol. 2020 May 19;16(5):e1007793. doi: 10.1371/journal.pcbi.1007793 (PMC7295246; doi:10.1371/journal.pcbi.1007793)
Supplement: S2 Table — Clinical physiological data of the four LUAD stages matched with the lncRNA and mRNA expression data. (PDF) [file pcbi.1007793.s011.pdf]

S2 Table. Distribution of clinical samples in gene expression data of LUAD.

	mRNA and lncRNA Data	microRNA Data	All samples
Control	59	46	85
Stage I	284	279	288
Stage II	122	122	124
Stage III	84	85	85
Stage IV	26	24	26
Total	575	556	608

The rows denoted as stages I, II, III, and IV represent the numbers of samples in the networks in the four stages of LUAD.
The row “control” gives the number of normal samples.
