# Supplementary material for: Dynamical network analysis reveals key microRNAs in progressive stages of lung cancer
Source: PLoS Comput Biol. 2020 May 19;16(5):e1007793. doi: 10.1371/journal.pcbi.1007793 (PMC7295246; doi:10.1371/journal.pcbi.1007793)
Supplement: S3 Table — Differentially expressed lncRNA, microRNA and mRNA profile data in the four stages of LUAD. (PDF) [file pcbi.1007793.s012.pdf]

S3 Table. The number of differential expression (DE) gene.

	DE lncRNAs			DE microRNAs			DE mRNAs		
	total	up	down	total	up	down	total	up	down
Stage I	104	56	48	112	24	88	1657	966	691
Stage II	118	75	43	114	28	86	1890	1077	813
Stage III	131	80	51	118	26	92	2039	1159	880
Stage IV	119	69	50	110	22	88	2016	1169	847

The threshold of the absolute value of $\log_2$ FC ($\log_2$ fold change) for filtering the three types of RNAs is set to be one, ensuring that their P-values are less than 0.05 in the differential expression analysis.
Fold changes greater than one correspond to up-regulated genes, while those less than minus one are associated with down-regulated genes. The rows Stages I, II, III, and IV represent the networks in the four stages of LUAD.
