# Supplementary material for: Dynamical network analysis reveals key microRNAs in progressive stages of lung cancer
Source: PLoS Comput Biol. 2020 May 19;16(5):e1007793. doi: 10.1371/journal.pcbi.1007793 (PMC7295246; doi:10.1371/journal.pcbi.1007793)
Supplement: S4 Table — The microRNA-mRNA and microRNA-lncRNA interactions relationships obtained by using the base of complementary pairing matching relationship data in the RNA sequences. (PDF) [file pcbi.1007793.s013.pdf]

S4 Table. The number of lncRNAs or mRNAs targeted by microRNAs and their relationships.

	DE lncRNA			DE miRNA			DE mRNA			miRNA-lncRNA	miRNA-mRNA
	total	up	down	total	up	down	total	up	down		
Stage I	77	44	33	58	12	46	728	456	272	711	3726
Stage II	89	59	30	57	13	44	840	514	326	765	3982
Stage III	99	61	38	61	13	48	944	564	380	826	4792
Stage IV	93	53	40	58	12	46	904	556	348	808	4376

The columns “total,” “up” and “down” represent the numbers of all, up-regulated and down-regulated RNAs in the four stages of LUAD.
The columns miRNA-lncRNA and miRNA-mRNA list the numbers of the edges in the microRNA-lncRNA and microRNA-mRNA subnetworks, respectively, in the networks of four stages of LUAD.
