# Supplementary material for: Dynamical network analysis reveals key microRNAs in progressive stages of lung cancer
Source: PLoS Comput Biol. 2020 May 19;16(5):e1007793. doi: 10.1371/journal.pcbi.1007793 (PMC7295246; doi:10.1371/journal.pcbi.1007793)
Supplement: S5 Table — The Pearson correlation of the RNA expression data between microRNA-mRNA and microRNA-lncRNA expressions is calculated. (PDF) [file pcbi.1007793.s014.pdf]

S5 Table. The number of lncRNAs or mRNAs selected by introducing negative correlation.

	DE lncRNA	DE miRNA	DE mRNA	miRNA-lncRNA	miRNA-mRNA
Stage I	56	58	480	202	1431
Stage II	59	55	519	206	1397
Stage III	70	61	595	276	1676
Stage IV	65	57	618	292	1653

The threshold of the correlation coefficient is set to be -0.1.
The columns of miRNA-lncRNA and miRNA-mRNA list the numbers of edges for the microRNA-lncRNA and microRNA-mRNA subnetworks, respectively, in the networks of four stages of LUAD. The rows stage I, II, III, and IV correspond to the networks of the four stages of LUAD.
