# Supplementary material for: Dynamical network analysis reveals key microRNAs in progressive stages of lung cancer
Source: PLoS Comput Biol. 2020 May 19;16(5):e1007793. doi: 10.1371/journal.pcbi.1007793 (PMC7295246; doi:10.1371/journal.pcbi.1007793)
Supplement: Attachment — Submitted filename: Response.pdf [file pcbi.1007793.s016.pdf]

March 6, 2020

Dear Dr. Ioshikhes and Dr. Lauffenburger:

Thank you very much for obtaining two referee reports on our manuscript **PCOMPBIOL-D-19-02215** entitled “Dynamical network analysis reveals key microRNAs in progressive stages of lung cancer.” We are very happy that Reviewer #1 evaluated our work quite positively and recommended publication in its present form, and Reviewer#2 provided a number of comments to improve the paper. In the re-submitted manuscript all the referee comments have been fully addressed. A detailed, point-by-point response to the comments is enclosed. The changes in the manuscript and supplementary materials are marked blue.

We wish to take this opportunity to thank both referees for their time and efforts to evaluate our work and also for their insightful comments that have resulted in an improved manuscript. We would also like to thank you for your professional handling of our paper. We hope our revised manuscript can be judged to have met the high standard of *PLOS Computational Biology*.

With kind regards,

Zi-Gang Huang, Professor
School of Life Science and Technology
Xi’an Jiao Tong University
Email: huangzg@xjtu.edu.cn
(on the behalf of all co-authors)

Point-by-point response to referee comments

Reviewer #1

General comments and recommendation: "*An important discovery in cancer research is the roles played by non-coding RNAs (ncRNAs) that have been identified as the oncogenic drivers and tumor suppressors in different cancer types. This has led to the fundamental competing endogenous RNA (CeRNA) hypothesis: microRNAs, mRNAs, and ncRNAs form an inseparable unity of RNA-level regulating network in the intracellular environment associated with cancer development, collectively known as the CeRNA network. In existing studies of CeRNA networks, a common approach was to identify some differentially expressed lncRNAs, mRNAs and microRNAs based on absolute fold changes and the values of the false positive ratio. A deficiency of this approach is that information used to construct the underlying CeRNA networks and to reveal the network functions with clinical implications is directly from data bases without consideration of any dynamical aspect. As a result, the reconstructed CeRNA networks are simply a combination of different kinds of RNAs, whereas the dynamical interplay and competition among different types of RNAs are completely ignored. However, cancer development and evolution are fundamentally a dynamical process. Reflected in the underlying CeRNA networks, it is unlikely that the intrinsic interactions, interplay and the network structure remain static during cancer development. To better understand cancer and to identify more effective biomarkers, the dynamical aspects of the CeRNA networks need to be studied. In the manuscript under review, the authors developed a dynamical network analysis to address this issue. Through this analysis, the authors uncovered ten specific microRNAs that can serve as effective biomarkers and prognostic tools for lung cancer.*

With available data from a gene database, e.g., RNA expression and clinical data of LUAD from the cancer genome atlas, the authors incorporated the time axis into the analysis by focusing on the four stages of LUAD progression to construct the lncRNA-microRNA-mRNA CeRNA network for each stage. The quantitative approach to reconstructing the CeRNA network consists of analyzing differentially expressed RNAs, matching microRNA targets by base complementary pairing, and selecting the negative correlation by invoking the CeRNA hypothesis. The authors then calculated the fold changes and the average expression level of RNAs in the CeRNA networks for the four stages and carried out Gene Ontology and Kyoto Encyclopedia of Genes and Genomes pathway enrichment analyses to validate the results. A finding is the emergence of two characteristically distinct types of networks that play an important role in the progression of LUAD from stage I to stage IV: one is common to all four stages, which the authors named as the common competing endogenous RNA network (CCEN), and another is unique for each stage, which the authors termed as the unique competing endogenous RNA networks (UCENs). Analyzing the properties of CCEN and UCENs, the authors uncovered a number of key genes that affect or even determine the survival of patients at each stage of LUAD: six microRNAs from the CCEN which affect the survival of LUAD patients at all stages, and four other microRNAs that influence the survival at each specific stage.

The work represents a timely contribution to cancer research because it has established a more comprehensive gene-data analysis framework than previous ones, not only providing a tool to probe more deeply into the mechanisms of cancer evolution than previously possible but also having the potential to lead to more effective biomarkers and drug targets for LUAD as well as other types of cancer. The paper is very well written. I recommend publication in its present form."

Response: We thank the referee for evaluating our work highly and for recommending publication in its present form.

Reviewer #2

General Comment: "*The authors construct and analyze CeRNA networks for four sequential stages of lung adenocarcinoma (LUAD) based on multi-omics data of long non-coding RNAs (lncRNAs), microRNAs and mRNAs. Their analysis shows the emergence of two characteristically distinct types of networks, common competing endogenous RNA network (CCEN) and unique competing endogenous RNA networks (UCENs), that play an important role in the progression of 4 different stage LUAD. The study yields some prognostic markers closely related to the survival of LUAD patients, and provides new insights into understanding cancer mechanisms and identifying targets for better drugs. The results are novel and interesting.*"

Response: We thank the referee for his/her positive evaluation of our work.

Comment 1: "*The authors study the CeRNA networks in four LUAD stages. The author should introduce some background on how these four stages are classified and what are the major differences among these four stages.*"

Response: We have provided a background description of the criteria for classifying the four stages of LUAD in **MATERIALS AND METHODS** on page 8, citing the original reference ([74]), which reads

- We follow the Tumor-Node-Metastasis (TNM) staging criteria for malignant tumors to classify LUAD into four stages. In particular, TNM is a standardized classification system established by the International Association for the Study of Lung Cancer to describe the development of lung cancer in terms of size and spread, where "T" describes the size of the tumor and any spread of the cancer into nearby tissues, "N" denotes the spread of the cancer to nearby lymph nodes, and "M" stands for metastasis, i.e., the spread of the cancer to other parts of the body [74]. For our datasets, we have that, of the remaining 608 samples, 288, 124, 85, and 26 are labeled as stages I, II, III, and IV, respectively, whereas 85 are normal. (More details about the classification criteria of LUAD can be found in Supplementary Note 2.)

In addition, In Supplementary Note 2, we have introduced more background information about LUAD stages and their major differences and listed the stage groupings for non-small-cell lung cancer in Supplementary Table 6. The new descriptions in Supplementary Note 2 are

- Lung Adenocarcinoma (LUAD) is a kind of lung cancer that is a malignant tumor. The International Association for the Study of Lung Cancer established the Lung Cancer Staging Project in 1998 and updated the Tumor-Node-Metastasis (TNM) classification system of malignant tumors,

which forms the basis for classifying lung cancer into distinct stages. The current (seventh) edition classifies all histotypes of lung cancer after evaluation of the outcomes from an extensive worldwide database of patients.

- TNM characterizes the size and location of the tumor, the location of cancer in the lymph nodes and to where the cancer has spread (Metastases). In particular, the letter "T" stands for primary tumor:

  "T1a" – tumor size $\leq$ 2cm;

  "T1b" – tumor size $> 2 \sim 3$cm;

  "T2a" – tumor size $> 3 \sim 5$cm;

  "T2b" – tumor size $> 5 \sim 7$cm;

  "T3" – tumor size $>$ 7cm and/or multiple tumor nodules in the same lobe;

  "T4" – multiple tumor nodules (of any size) in the same lung but in a different lobe.

  The letter "N" denotes the spread of the cancer to nearby lymph nodes:

  "N0" – no regional lymph node metastasis;

  "N1" – metastasis in ipsilateral peribronchial and/or ipsilateral hilar lymph nodes as well as intrapulmonary nodes including involvement by direct extension;

  "N2" – metastasis in ipsilateral mediastinal and/or subcarinal lymph node(s);

  "N3" – metastasis in contralateral mediastinal, contralateral hilar, ipsilateral or contralateral s-calene, or supraclavicular lymph node(s).

  The letter "M" describes distant metastasis:

  "M1a" – malignant pleural or pericardial effusions and/or separate tumor nodules in the contralateral lung;

  "M2b" – distant metastasis in extrathoracic organs.

Based on the seventh edition of the TNM classification system described above, we list the stage groupings for non-small-cell lung cancer in Supplementary Table 6. In order to have sufficient data amount for statistical analysis of each LUAD stage, in our work we combine stage Ia and Ib and label them as stage I, and do the same for Stages II, III, and IV.

Comment 2: "*In figure 2, blue dots represent Gene expression values and yellow dots represent differential expression analysis results. However, it is not clear what does the legend "Retention" and "Discard" means in the figure. It is not described neither in the figure caption nor in the main text. The author should give explanations to it.*"

Response: We have provided a detailed explanation in the caption of Fig. 2, which reads

- The blue group “Retention” represents RNAs selected through analyzing differentially expressed (DE) RNAs, matching microRNA targets, and selecting the negative correlation in the CeRNA networks. For comparison, the yellow group “Discard” describes RNAs processed only through the differential expression analysis, which are not used in the construction of the CeRNA networks. The hypothesis tests between the “Discard” and “Retention” groups are of the Wilcoxon type. A. Gene expression values of lncRNAs, microRNAs and mRNAs in the reconstructed CeRNA networks of the four stages of LUAD. B. The corresponding fold-change values of lncRNAs, microRNAs and mRNAs.

Comment 3: “*Since the study have utilized statistical tests and obtained P-values for different tests, the authors should describe what type of tests are used during different analyses in the Methods section.*”

Response: In our work, we have used four types of statistical analysis with hypothesis tests to obtain the P-values. In the revised manuscript, we have added a clarification about the P-values in **MATERIALS AND METHODS** [near Eq. (1) on page 9], which reads

- Gene enrichment analysis is a widely used approach to identifying biological connections. In Figs. 3 and 5, we implement the hypergeometric model to assess whether the number of selected genes in the CeRNA networks associated with lung cancer is larger than that which can be expected purely by chance. In particular, the P-value determines whether any term annotates a specified list of genes at frequency greater than that which can be expected by chance, as determined by the hypergeometric distribution:

$$p = 1 - \sum_{i=0}^{k-1} \frac{\binom{N-M}{n-i}\binom{M}{i}}{\binom{N}{n}}, \tag{1}$$

  where N is the total number of genes in the background distribution, M is the number of genes within that distribution that are annotated (either directly or indirectly) to the node of interest, n is the size of the list of genes of interest, and k is the number of genes within that list which are annotated to the node. The background distribution by default is all the genes that have an annotation.

A brief note has also been added in the captions of Figs. 3, 5, 7 and 8. In addition, we have provided the following explanation in Supplementary Note 3:

- In our construction of the CeRNA network, RNAs are processed through a differentially expressed analysis, where the basic statistic used for significance assessment is the moderated t-statistic [1], which is computed for each contrast. This has the same interpretation as an ordinary t-statistic except that the standard errors have been moderated across genes, i.e., squeezed towards a common value, using a simple Bayesian model. This has the effect of borrowing information from the ensemble of genes to enhance inference about each individual gene. Moderated t-statistics lead to P-values in the same way that ordinary t-statistics do except that the degrees of freedom are increased, reflecting the greater reliability associated with the smoothed standard errors.

- The two-sample Wilcoxon test (also known as "Mann-Whitney" test) [2,3] is applied to vectors of the RNA expression data, with the following notations for statistical significance: ns – $p > 0.05$; * – $p \leq 0.05$; ** – $p \leq 0.01$; *** – $p \leq 0.001$; **** – $p \leq 0.0001$.

With three references. The Wilcoxon test has also been mentioned in the caption of Fig. 2 in the main text.

At the end of the subsection entitled "Framework of analysis" on page 10 in the main text, we have added

- Log-rank tests are used to carry out a univariate analysis of the Kaplan-Meier survival curve, which belong to the chi-square test, where all time points of the sample survival information are equal (i.e., with weight setting to one).

This has also been mentioned in the captions of Figs. 7 and 8.

Comment 4: "*The authors calculate the average expression levels of RNAs and CeRNA networks. The authors should also provide a few individual behaviors in the Supplementary Material to show how different is the individual behavior from the group average.*"

Response: Fold Change characterizes the relative expression level of interested samples to that of the control samples. For $\log_2$ Fold-Change $= 1$, the individual RNA expression level equals the group average of RNAs. The individual RNA behavior can be assessed from the volcano map of RNA expression. We have provided the volcano maps for the expressions of lncRNA, microRNA and mRNA at the four LUAD stages (new Supplementary Note 4 and Supplementary Figs. 5-7).

We have provided a small paragraph discussing the individual RNA behaviors (near the top of page 9).

Comment 5: "*It would be instructive to do some additional surrogate test to prove the results are physiologically meaningful. For example, the authors could make a CeRNA network consists of RNAs from different subjects, or to compare networks from real data with those from noisy/artificial processes, and check whether reported behavior is statistically different from the surrogate tests.*"

Response: Surrogate test is useful for testing the effects of different data sources on the results. However, our work is to analyze the CeRNA network constructed from genomics data collected from TCGA and clinical data from a large number of LUAD samples in a step-by-step manner. If we use surrogate test to obtain paired signals from different LUAD samples to construct the CeRNA network, there will be many possible CeRNA networks, making it extremely difficult (if not impossible) to determine the networks for identifying biomarkers. Another aspect of our work is a focus on the variations among the progressive stages of lung cancer, and the number of sequential CeRNA networks constructed for different LUAD stages is limited. Our approach is then to use the gene expression, the FC values of the CeRNA network nodes, and gene enrichment analysis to validate the network. For surrogate networks, the meanings of these tests are not clear. We have demonstrated that our approach does lead to a number of microRNA biomarkers, and we have carried out indirect validation to test the effectiveness of the targets, which includes the Kaplan-Meier survival analysis based on combining

the time event correspondence to the clinical physiological data of the sample patient and *a priori* knowledge or expert system verification.

This discussion has been added to the main text (the second last paragraph in the section of DISCUSSION).

Comment 6: “*The authors studied the CeRNA networks in four LUAD stages and find the emergence of two characteristically distinct types of networks play important roles in the progression of 4 different stage LUAD. The findings of transitions in CeRNA networks with progression of the stage/disease is reminiscent of transitions and reorganization in network of physiological /organ systems interaction in the human body at larger space/time scales, reported in earlier studies within the broader context of the field of Network Physiology...... It would be instructive if the authors make parallels and discuss their results within the framework/context of Network Physiology, and the above works.*”

Response: We thank the referee for suggesting the five references. We have read the papers and cited them in the revised manuscript with discussions. In particular, in the last paragraph in INTRODUCTION, we have written

- We hold the belief that cancer development and evolution are fundamentally a dynamic process. Manifested in the underlying CeRNA networks, it is unlikely that the intrinsic interactions, interplay and the network structure remain static during cancer development. To better understand cancer and to identify more effective biomarkers, the dynamical aspects of the CeRNA networks must be taken into account. This is in line with the field of network physiology [23-27], where transitions and reorganization occur in the networks of physiological/organ systems in the human body on larger spatial and temporal scales, which can be constructed using continuous time series. In the CeRNA network analysis, it is infeasible to collect RNA data in continuous time. Nonetheless, with the presently available gene data, we are able to incorporate the time axis into the analysis but only for a limited set of time intervals.

In DISCUSSION, we have added the following (the seventh paragraph):

- The emerging field of network physiology and medicine [23-27] focuses on the associations between network structures and physiologic states, which relies heavily on measurements from biomedical experiments on large spatial and temporal scales. In this sense, our findings demonstrate an association between CeRNA network and physiologic dysfunction of the lung organ.
